# Involvement of the ERK pathway in the protective effects of glycyrrhizic acid against the MPP^+^-induced apoptosis of dopaminergic neuronal cells

**DOI:** 10.3892/ijmm.2014.1830

**Published:** 2014-07-02

**Authors:** LESHENG TENG, CHUNJIA KOU, CHENGYU LU, JIAMING XU, JING XIE, JIAHUI LU, YAN LIU, ZHENZUO WANG, DI WANG

**Affiliations:** 1 College of Life Science, Jilin University, Changchun, Jilin 130012, P.R. China; 2 College of Clinical Medicine, Jilin University, Changchun, Jilin 130012, P.R. China; 3 College of Electronic Science and Engineering, Jilin University, Changchun, Jilin 130012, P.R. China

**Keywords:** glycyrrhizic acid, 1-methyl-4-phenylpyridinium, apoptosis, extracellular signal-regulated kinase, mitochondria

## Abstract

Glycyrrhizic acid (GA), a major compound separated from Radix Glycyrrhizae, has been shwon to exert various biochemical effects, including neuroprotective effects. In the present study, we investigated the protective effects of GA against 1-methyl-4-phenylpyridinium (MPP^+^)-induced damage to differentiated PC12 (DPC12) cells. Compared with the MPP^+^-treated cells, GA markedly improved cell viability, restored mitochondrial dysfunction, suppressed the overexpression of cleaved poly(ADP-ribose) polymerase (PARP), and suppressed the overproduction of lactate dehydrogenase (LDH) and intracellular Ca^2+^ overload. The protective effects of GA on cell survival were further confirmed in primary cortical neurons. GA markedly increased the expression of phosphorylated extracellular signal-regulated kinase (p-ERK), as well as its migration from the cytoplasm to nucleus. PD98059, an inhibitor of ERK, blocked GA-enhanced ERK activation and reduced cell viability. However, pre-treatment with GA had no effects on the expression of phosphorylated AKT (p-AKT) and total AKT (t-AKT). These results indicate that the GA-mediated neuroprotective effects are associated with its modulation of multiple anti-apoptotic and pro-apoptotic factors, particularly the ERK signaling pathway. This study provides evidence supporting the use of GA as a potential therapeutic agent for the treatment of neurodegenerative diseases and neuronal injury.

## Introduction

Parkinson’s disease (PD) is a recognized progressive neurodegenerative disorder which is caused by a decrease in the number of dopaminergic neurons located in the substantia nigra (1,2). Although the etiology of the disease remains unknown, postmortem studies have indicated that dying cells not only bear signs of necrosis, but also of apoptosis, in particular chromatin condensation, DNA fragmentation, oxidative damage, mitochondrial dysfunction and caspase activation (3,4). 1-Methyl-4-phenylpyridinium (MPP^+^) is commonly used *in vitro* and *in vivo* to produce models of PD (5,6). Similar to other dopaminergic toxins, MPP^+^ causes oxidative stress and the selective death of dopaminerigic neuronal cells, such as PC12 (7) and SH-SY5Y cells (8).

A number of studies have reported that herbal preparations and their natural compounds display broad protective effects against neurotoxicity in various neurodegenerative diseases (9,10). Glycyrrhizic acid (GA), a major active ingredient separated from Radix Glycyrrhizae, possesses anti-inflammatory and anti-viral effects (11,12). It has been well documented that GA exerts marked neuroprotective effects against 6-hydroxydopamine- or glutamate-induced damage to neuronal cells (13,14). However, the contribution of GA toward MPP^+^-induced cell damage and the underlying mechanisms have not yet been fully elucidated.

It is well known that extracellular signal-regulated kinase (ERK) plays a key role in cell proliferation, differentiation, survival and apoptosis (15). The phosphorylation of ERK has been shown to be critical for mediating the neuroprotectives effects of leptin (16). Combined with the activation of ERK, mitochondrial depolarization is associated with apoptotic cell death (17). Another pathway involved in this process is the PI3K/AKT signaling pathway, which is essential for rescuing neuronal cells from oxidative stress (18).

We therefore hypothesized that GA exerts neuroprotective effects against MPP^+^-induced cell damage. To examine this hypothesis, in this study, we investigated the inhibitory effects of GA on MPP^+^ cytotoxicity and the underlying mechanisms. Our data revealed that GA attenuated MPP^+^-induced cell death, the high apoptotic rate, the intracellular Ca^2+^ overload, the overproduction of lactate dehydrogenase (LDH), as well as mitochondrial dysfunction. Further experiments indicated that the activation of ERK contributes to the GA-mediated neuroprotection of dopaminergic neuronal cells.

## Materials and methods

### Cell culture

PC12 cells (rat adrenal gland pheochromocytoma cells; obtained from ATCC, Manassas, VA, USA; CRL-1721 passages <10) were maintained as monolayer cultures in Dulbecco’s modified Eagle’s medium (DMEM) supplemented with 10% horse serum (HS), 5% fetal bovine serum (FBS) and penicillin (100 IU/ml), and streptomycin (100 μg/ml) (all from Invitrogen, Carlsbad, CA, USA), under a humidified atmosphere containing 5/95% CO_2_/air at 37 °C. The culture medium was changed every 3 days. The PC12 cells were treated with 20 ng/ml nerve growth factor (NGF; Sigma-Aldrich, St. Louis, MO, USA) in DMEM supplemented with 1% FBS and 1% HS and incubated for 72 h to induce differentiation.

Primary cultures of neurons were prepared from fetal cortices of pregnant Sprague-Dawley rats [embryonic days (E) 17–18] as previously described (19). Briefly, the neurons were dissociated from the cerebral cortex of embryonic rats and were plated in 96-well culture plates which had been previously coated with poly-D-lysine (Invitrogen). The cells were maintained in neurobasal medium supplemented with 2% B27 and 1% GlutaMAX (both from Invitrogen). The purity of the primary cortical neuronal cells was 88.1±5.6% (Fig. 2A) which was determined by β3-tubulin (red fluorescence) staining using ImageJ software.

### Analysis of cell viability and cellular morphology

Cell viability was measured by a quantitative colorimetric assay with 3-(4,5-dimethylthiazol-2-yl)-2,5-diphenyltetrazolium bromide (MTT; Sigma-Aldrich) as described in a previous study (20). Briefly, the differentiated PC12 (DPC12) cells and primary neurons were seeded into 96-well plates at 5×10^4^ cells per well. The cells were pre-treated with 5–100 μM GA for 3 h, co-treated with 0.5 or 4 mM MPP^+^ for 24 h, and then incubated with MTT solution (5 mg/ml) for a further 4 h. A total of 100 μl dimethyl sulfoxide (DMSO) was added to each well and then the absorbance was measured using a microplate reader (Bio-Rad, Berkeley, CA, USA) at 540 nm. Cell viability was expressed as a percentage of the value in the control group (untreated cells). Prior to MTT assay, the morphology of the primary neurons was detected by normal photography (10′, Axio Observer Z1; Carl Zeiss, Inc., Oberkochen, Germany).

### Measurement of LDH release

The release of LDH into the culture medium was measured using an *In Vitro* Toxicology Assay kit (Sigma-Aldrich). Briefly, the PC12 cells were seeded into 96-well plates at 2×10^4^ cells per well. Following differentiation, the cells were pre-treated with 25 μM GA for 3 h and co-treated with 4 mM MPP^+^ for 24 h. A total of 60 μl mixed assay solution was added into 30 μl culture medium collected from each group. Following 30 min of incubation at room temperature in the dark, 10 μl 1 N HCl were added to terminate the reaction. The absorbance was measured at a wavelength of 490 nm using a microplate reader (Bio-Rad). The values of the treated cells were expressed as a percentage of the corresponding untreated cells.

### Mitochondrial membrane potential (MMP) analysis

5,5′,6,6′-Tetrachloro-1,1′,3,3′ tetraethylbenzimidazolylcarbocyanine iodide (JC-1; Sigma-Aldrich) was used to measure changes in MMP. The PC12 cells (1×10^5^) were seeded in 12-well plates and allowed to differentiate. The cells were pre-treated with 25 μM GA for 3 h and co-treated with 4 mM MPP^+^ for 24 h. The treated DPC12 cells were incubated with 2 μM JC-1 at 37°C for 10 min. After 3 washes with phosphate-buffered saline (PBS), the changes in MMP of the DPC12 cells were analyzed using a fluorescence microscope (×20 magnification; Axio Observer Z1, CCD camera; Carl Zeiss, Inc.).

### Measurement of intracellular Ca^2+^ concentration

Fluo-4 AM (Invitrogen) staining was used to measure the intracellular Ca^2+^ concentration. The DPC12 cells were pre-treated with 25 μM GA for 3 h and then co-treated with 4 mM MPP^+^ for a further 3 h. The cells were incubated with Fluo-4 AM (final concentration, 5 μM) at 37°C for 30 min, and washed 3 times to remove the excess probe. The fluorescence intensity was determined using a fluorescence microscope (×40 magnification; Axio Observer Z1, CCD camera; Carl Zeiss). The experiment was repeated 3 times and the average fluorescence intensity for each cell was calculated using ImageJ software.

### Flow cytometric analysis of apoptosis

Flow cytometric analysis was used to assess the membrane and nuclear events during apoptosis, as previously described (7). The assay was performed with a two-color analysis of fluorescein isothiocyanate (FITC)-labeled Annexin V binding propidium iodide (PI) (Becton-Dickinson Co., Miami, FL, USA). The DPC12 cells were pre-treated with 25 μM GA for 3 h and then co-treated with 4 mM MPP^+^ for a further 12 h. The cells were suspended (1×10^6^/ml) in binding buffer and incubated for 10 min with 5 μl Anexin V-FITC (20 μg/ml) and 10 μl PI (50 μg/ml) at room temperature in the dark. The level of fluorescence was analyzed using a flow cytometer (Cytomics™ FC 500; Beckman Coulter, Inc., Brea, CA, USA). The experiment was repeated 3 times.

### Western blot analysis

The treated cells were lysed with RIPA buffer containing 1% protease inhibitor cocktails and 2% phenylmethanesulfonyl fluoride (PMSF) (all from Sigma-Aldrich). For the detection of ERK translocation, the preparation of cytoplasmic and nuclear extracts was carried out as described in a previous study by Yang *et al* (21). After the supernatant collection, the protein concentration was determined using the Bradford method. Proteins were separated on a 10% SDS-PAGE gel and transferred electrophoretically onto nitrocellulose membranes (Bio Basic, Inc., Markham, Ontario, Canada). The transferred membranes were then blotted with primary antibodies (dilution of 1:1,000) to: phosphorylated ERK (p-ERKs), total ERK (t-ERKs), phosphorylated AKT (p-AKT), total AKT (t-AKT), cleaved poly(ADP-ribose) polymerase (PARP), glyceraldehyde 3-phosphate dehydrogenase (GAPDH) and lamin B (Cell Signaling Technology, Inc., Danvers, MA, USA) at 4°C overnight, followed by treatment with horseradish peroxidase-conjugated secondary antibodies (Santa Cruz Biotechnology, Inc., Dallas, TX, USA). Chemiluminescence was detected using ECL detection kits (GE Healthcare, Buckinghamshire, UK). The intensity of the bands was quantified by scanning densitometry using Quantity One 4.5.0 software (Bio Basic, Inc.).

### Statistical analysis

Data are expressed as the means ± SD. One-way ANOVA was applied to determine the statistical significance, followed by post-hoc multiple comparisons (Dunn’s test). A value of P<0.05 was considered to indicate a statistically significant difference.

## Results

### Effects of GA on cell viability, LDH release and apoptosis of DPC12 cells or primary cortical neurons

**Figure 1:**
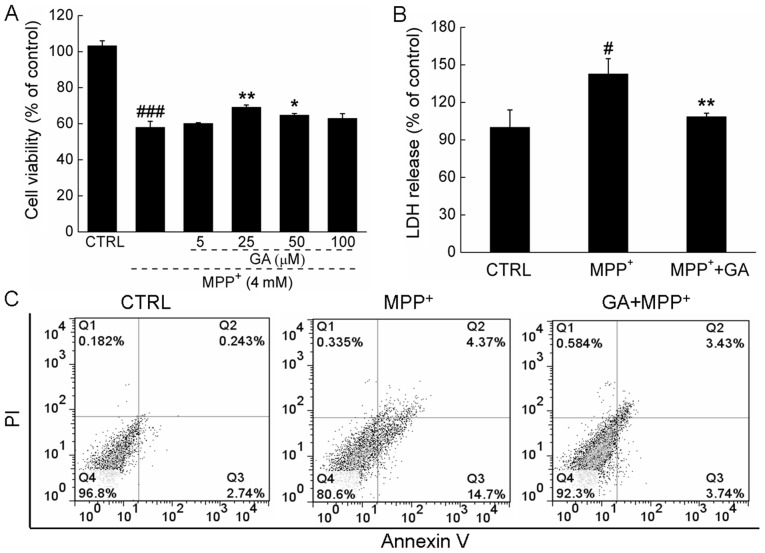
Effects of glycyrrhizic acid (GA) against 1-methyl-4-phenylpyridinium (MPP^+^)-induced neurotoxicity in differentiated PC12 (DPC12) cells. Cells were pre-treated with 5–100 μM GA for 3 h, followed by exposure to 4 mM MPP^+^ for 24 h. Compared with the MPP^+^-treated cells, pre-treatment with GA (A) enhanced cell viability, (B) reduced the release of intracellular LDH and (C) decreased the apoptotic rate. Data are expressed as a percentage of the corresponding control cells and are presented as the means ± SD (n=3). ^#^P<0.05, ^###^P<0.001 vs. untreated cells; ^*^P<0.05 and ^**^P<0.01 vs. MPP^+^-exposed cells. CTRL, control.

**Figure 2:**
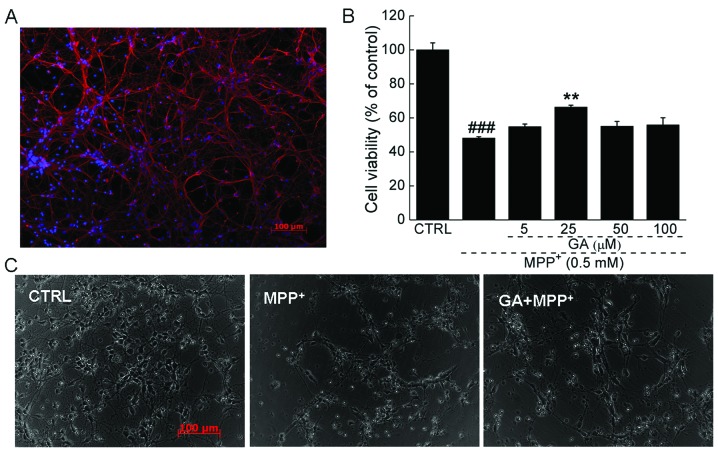
Glycyrrhizic acid (GA) exerts protective effects on primary cortical neuronal cell survival against 1-methyl-4-phenylpyridinium (MPP^+^) neurotoxicity. (A) The purity of primary cortical neuronal cells was determined by β3-tubulin staining. Cells were pre-treated with 5–100 μM GA for 3 h, followed by exposure to 0.5 mM MPP^+^ for 24 h. Pre-treatment with GA (B) promoted cell viability and (C) prevented the morphological changes induced by MPP^+^. Data are expressed as a percentage of the corresponding control cells and are presented as the means±SD (n=6). ^###^P<0.001 vs. untreated cells; ^**^P<0.01 vs. MPP^+^-exposed cells. CTRL, control.

Exposure to 4 mM MPP^+^ for 24 h resulted in 41.9±3.3% cell death compared with the control cells; however, pre-treatment with 25 μM GA significantly attenuated the MPP^+^-induced cytotoxicity and increased cell viability (58.1±3.3 vs. 69.2±1.3%; P<0.01; Fig. 1A). The protective effects of GA (25 μM) on cell viability were further confirmed in primary neurons (48.2±0.8 vs. 65.3±1.1%; P<0.01; Fig. 2B). Pre-treatment with 25 μM GA for 3 h following exposure to 4 mM MPP^+^ for 24 h markedly suppressed the overproduction of LDH induced by MPP^+^ in the DPC12 cells (108.4±2.9 vs. 142.7±12.2%; P<0.01; Fig. 1B). Moreover, double-staining with Annexin V-FITC and PI was used to detect the apoptotic rate of DPC12 cells following treatment. The increase in the apoptotic rate of the MPP^+^-exposed cells was reversed by pre-treatment with 25 μM GA (7.2 vs. 19.1%; Fig. 1C). The morphological changes in the primary cortical neurons were visualized by phase-contrast imaging. Compared with the untreated cells, shrinkage and detachment were observed following exposure to MPP^+^. Pre-treatment with 25 μM GA markedly reversed the morphological damage caused by MPP^+^ (Fig. 2C).

### Effects of GA on mitochondrial dysfunction, intracellular Ca^2+^ overload and the expression of cleaved PARP

**Figure 3:**
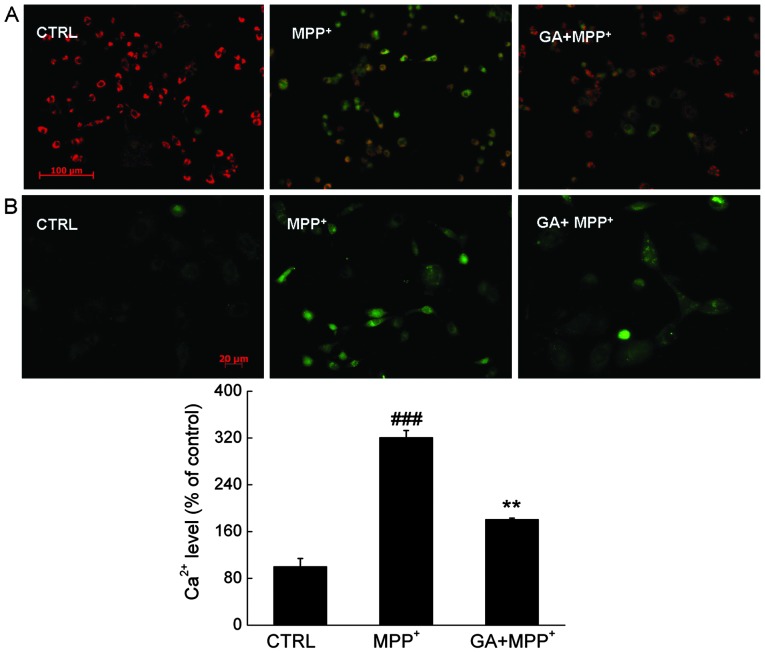
Effects of glycyrrhizic acid (GA) on (A) mitochondrial dysfunction (×20 magnification; scale bar, 100 μm) and (B) intracellular Ca^2+^ overload (×40 magnification; scale bar, 20 μm) induced by 1-methyl-4-phenylpyridinium (MPP^+^). Cells were pre-treated with 25 μM GA for 3 h, followed by exposure to 4 mM MPP^+^ for (A) 12 or (B) 3 h. Data are expressed as a percentage of the corresponding control cells and are presented as the means ± SD (n=3). ^###^P<0.001 vs. untreated cells, ^**^P<0.01 vs. MPP^+^-exposed cells. CTRL, control.

MMP related to mitochondrial permeability plays an important role in the cell apoptotic pathway. Compared with the MPP^+^-exposed cells, after 12 h of co-treatment, 25 μM GA restored the dissipation of MMP indicated by an increase in the emission of red fluorescence (Fig. 3A).

The results from Fluo-4 AM staining showed that after 3 h of incubation of the PC12 cells, GA mitigated the calcium overload caused by MPP^+^, indicated by a reduction in the emission of green fluorescence (Fig. 3B).

**Figure 4:**
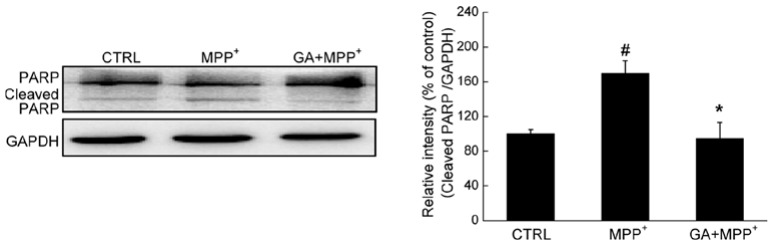
Glycyrrhizic acid (GA) suppressed the high expression of cleaved PARP caused by 1-methyl-4-phenylpyridinium (MPP^+^). Differentiated PC12 (DPC12) cells were pre-treated with GA for 3 h and then co-treated with 4 mM MPP^+^ for 24 h. Data are expressed as a percentage of the corresponding control cells and are presented as the means ± SD (n=3). ^#^P<0.05 vs. untreated cells; ^*^P<0.05 vs. MPP^+^-exposed cells. CTRL, control.

Furthermore, the level of cleaved PARP, a hallmark of apoptosis, was determined by western blot analysis. A significant enhancement in the expression of cleaved PARP was observed in the DPC12 cells exposed to 4 mM MPP^+^ for 24 h. Conversely, pre-treatment with 25 μM GA reduced the overexpression of cleaved PARP (P<0.05; Fig. 4).

### The ERK, but not the AKT pathway contributes to the GA-mediated neuroprotective effects

**Figure 5:**
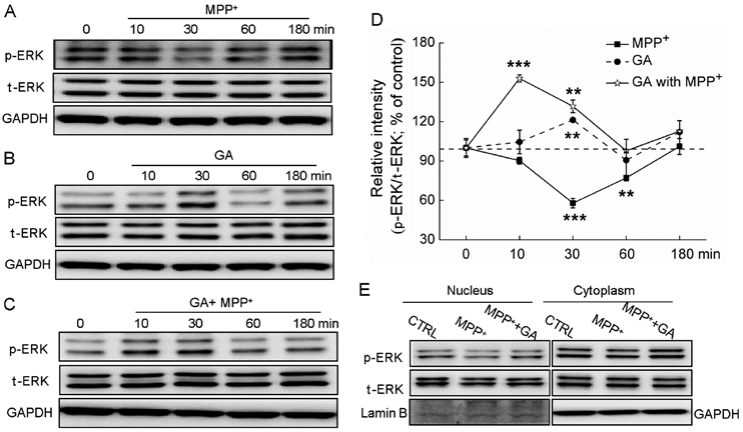
The extracellular signal-regulated kinase (ERK) signaling pathway contributes to the glycyrrhizic acid (GA)-mediated neuroprotective effects against 1-methyl-4-phenylpyridinium (MPP^+^) neurotoxicity. (A) Differentiated PC12 (DPC12) cells were treated with 4 mM MPP^+^ and collected at 0, 10, 30, 60 and 180 min. (B) DPC12 cells were pre-treated with 25μM GA for 3 h, and then collected at 0, 10, 30, 60 and 180 min. (C) Following pre-treatment with 25 μM GA for 3 h, cells were collected at 0, 10, 30, 60 and 180 min after exposure to 4 mM MPP^+^. (D) Quantification data of the expression of phosphorylated ERK (p-ERK) were normalized to the corresponding values of total ERK (t-ERK) and expressed as a percentage of corresponding cells collected at 0 min. (E) GA enhanced the migration of p-ERK from the cytoplasm to the nucleus. DPC12 cells were pre-treated with or without 25 μM GA for 3 h and then co-treated with MPP^+^ for 1 h. Data are expressed as the means ± SD (n=3) and analyzed by one-way ANOVA. ^**^P<0.01 and ^***^P<0.001 vs. non-treated cells. CTRL, control.

Exposure to MPP^+^ significantly suppressed the expression of p-ERK, but not that of t-ERK (Fig. 5A). Treatment with GA (25 μM) alone increased the phosphorylation of ERK after 30 min of incubation (P<0.01; Fig. 5B).Pre-treatment with GA (25 μM) markedly reversed the decrease in the expression of p-ERK caused by MPP^+^ at 10 and 30 min of treatment (Fig. 5C). Furthermore, the translocation of activated ERK from the cytoplasm to the nucleus was determined. Following exposure to 4 mM MPP^+^ for 60 min, the expression of p-ERK in the nucleus was reduced; by contrast, pre-treatment with GA for 3 h significantly reversed the MPP^+^-induced suppression of the translocation of ERK to the nucleus (Fig. 5E).

**Figure 6:**
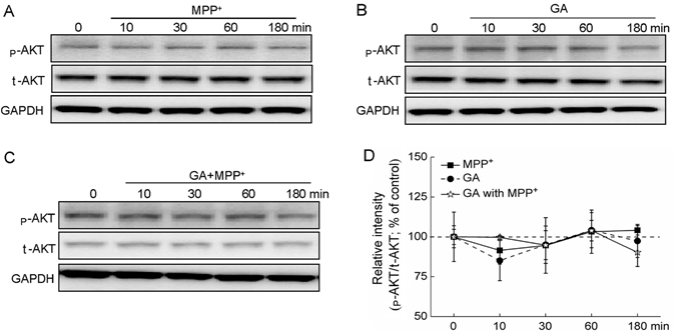
AKT signaling pathway is not involved in the glycyrrhizic acid (GA)-mediated neuroprotective effects against 1-methyl-4-phenylpyridinium (MPP^+^). (A) Differentiated PC12 (DPC12) cells were collected at 0, 10, 30, 60 and 180 min after exposure to 4 mM MPP^+^. (B) DPC12 cells were pre-treated 25 μM GA for 3 h, and then collected at 0, 10, 30, 60 and 180 min. (C) Following pre-treatment with 25 μM GA for 3 h, cells were collected at 0, 10, 30, 60 and 180 min after exposure to 4 mM MPP^+^. (D) Quantification data of the expression of phosphorylated AKT (p-AKT) were normalized to the corresponding values of total AKT (t-AKT) and expressed as a percentage of corresponding cells collected at 0 min. Data are the means ± SD of 3 replicate values in 3 separate experiments and analyzed by one-way ANOVA.

Additionally, treatment with MPP^+^ alone, GA alone and MPP^+^ plus GA had no effects on the expression of p-AKT and t-AKT (Fig. 6A–D).

**Figure 7:**
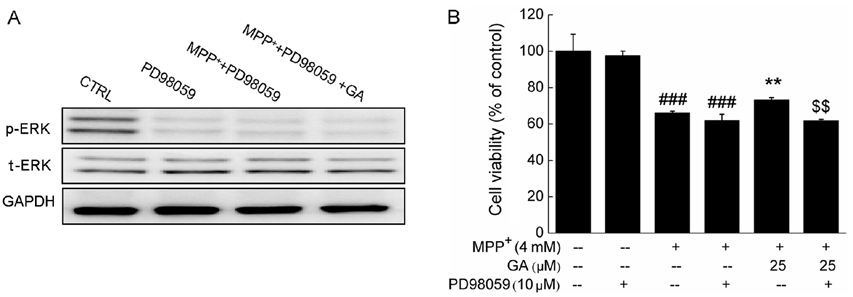
Promoting effects of glycyrrhizic acid (GA) on (A) the phosphorylaiton of extracellular signal-regulated kinase (ERK) and (B) cell viability were blocked by PD98059 (ERK inhibitor). Cells were pre-treated with or without 10 μM PD98059 for 30 min, and then exposed to 25 μM GA for 3 h and incubated with 4 mM 1-methyl-4-phenylpyridinium (MPP^+^) for (A) 1 or (B) 24 h. Data are expressed as the means ± SD (n=3) and analyzed using one-way ANOVA. ^###^P<0.001 vs. control group; ^**^P<0.01 vs. MPP^+^-treated group; ^$$^P<0.01 vs. GA-treated group. CTRL, control.

Further experiments revealed that the GA-induced increase in the phosphorylation of ERK and the improvement in cell viability were markedly abrogated by pre-treatment with 10 μM PD98059, an ERK inhibitor (Fig. 7A and B). Collectively, our data suggest that the ERK, but not the AKT signaling pathway contributes to the GA-mediated neuroprotective effects against MPP^+^-induced DPC12 cell damage.

## Discussion

PC12 cells, which possess a dopamine synthesis, metabolism and transporting system (22), can shift their phenotype, changing from proliferating, undifferentiated cells into post-mitotic, differentiated, neurite-bearing neurons following incubation with NGF (23). Studies have demonstrated that DPC12 cells are more sensitive to neurotoxins (24). The present study clearly confirms that GA exerts marked neuroprotective effects, as evidenced by our results: GA greatly ameliorated the MPP^+^-induced reduction in cell viability, the increased apoptotic rate, the intracellular Ca^2+^ overload and the dissipation in MMP in the DPC12 cells. GA also exerted suppressive effects on the overproduction of intracellular LDH caused by MPP^+^. Consistent with the results of previous studies (25,26), this suppressive effect on the LDH level is a consequence of the protective effects of GA against neurotoxicity. PARP is a family of proteins involved in a number of cellular processes, including DNA repair and programmed cell death (27). GA markedly suppressed the enhanced expression of cleaved PARP caused by MPP^+^.

The pro-survival activation of ERK is well known in dopaminergic neurons (16,28). The phosphorylation of ERK in SH-SY5Y cells has been shown to be suppressed after 4 h of exposure to MPP^+^ (29). Our study confirmed that MPP^+^ reduced the phosporylation of ERK; by contrast, GA reversed the MPP^+^-mediated inhibition of ERK activation. Additionally, the presence of PD98059 eradicated the effects of GA on ERK activation and protection of cell viability. Compared with the MPP^+^-treated cells, treatment with GA resulted in an increase in p-ERK nuclear migration. It has previously been demonstrated that activated ERK migrates to the nucleus where it regulates transcription factors, leading to changes in gene expression and cell proliferation (30). Collectively, our data indicate that the ERK signaling pathway contributes to the GA-mediated protective effects against MPP^+^-induced DPC12 cell damage.

Our data further indicated that pre-treatment with GA markedly blocked the calcium influx caused by MPP^+^. Excessive cytosolic calcium causes a wide range of subcellular pathological responses, in particular the dysfunction of mitochondrial membrane permeability (31). Growing experimental evidence suggests that the mitochondrial-dependent pathway plays a central role in cell apoptosis (17). A key feature of mitochondrial apoptosis is the disruption of the membrane potential, mainly caused by increased membrane permeability (32). The present study demonstrated that GA restored the dissipation of MMP and promoted the activation of ERK. As reported previously, the activation of ERK regulates mitochondrial function (33,34). Previous studies have demonstrated that ERK inhibitors downregulate the expression of B-cell lymphoma-2 (Bcl-2) and Bcl-extra large (Bcl-xL) (33), which are located in the outer membrane of the mitochondria and regulate mitochondrial function (35). However, the association between the activation of ERK and mitochondrial function and their involvement in the GA-mediated neuroprotective effects require further investigation.

It has been demonstrated that GA exerts neuroprotective effects against 6-hydroxydopamine-induced cytotoxicity in PC12 cells via PI3K/AKT pathway (13); however, in our study, we did not observe any significant effects on the activation of AKT following treatment with GA or MPP^+^. In another separate experiment, GA did not exert any effects on the glutamate-induced decrease in the level of p-AKT. The different results noted in our study may due to the different microenvironmental system. Furthermore, the accumulation of cells in the G1 phase is considered one of the factors responsible for MPP^+^-induced cell damage (36). In our study, MPP^+^-induced G1 phase arrest was observed in the DPC12 cells; however, pre-treatment with GA failed to reverse this effect (data not shown).

In conclusion, our data demonstrate that GA exerts significant protective effects on neuronal cells against MPP^+^ neurotoxicity, as indicated by the suppression of the intracellular Ca^2+^ overload, the restoration of mitochondrial dysfunction, and the increase in the expression and migration of p-ERK. The present findings provide pharmacological evidence to support the therapeutic application of GA in the treatment of neurodegenerative diseases.
